# Analysis of Throughput and Delay for an Underwater Multi-DATA Train Protocol with Multi-RTS Reception and Block ACK

**DOI:** 10.3390/s20226473

**Published:** 2020-11-12

**Authors:** Ho Young Hwang

**Affiliations:** School of Computer and Information Engineering, Kwangwoon University, Seoul 01897, Korea; hyhwang@kw.ac.kr; Tel.: +82-2-940-8265

**Keywords:** underwater wireless sensor networks, underwater acoustic propagation delay, medium access control protocol

## Abstract

We propose an underwater multi-DATA train protocol with multi-RTS reception and block ACK (BACK) for underwater acoustic sensor networks. Due to long underwater acoustic propagation delay, some RTS frames may not overlap at a sink node, even if the RTS frames were sent to the sink node simultaneously by different sensor nodes. We consider that our underwater sink node can recover these nonoverlapping RTS frames. Since our RTS frame contains ID of the RTS sending node and a timestamp, the sink node calculates the propagation delay between the RTS sending node and the sink node, then broadcasts a CTS frame. Since our CTS frame contains when each RTS sending node can transmit a DATA frame to the sink node, multiple DATA frames transmitted by different sensor nodes can be formed as a train at the sink node. We also propose an underwater BACK protocol which is analogous to our proposed underwater multi-DATA train protocol. We analyze normalized throughput and mean access delay of our proposed protocols and the conventional protocols. The analytical and simulation results show that our analysis is accurate and our proposed protocols outperform the conventional protocols. Our proposed protocol can shorten the delay and increase the throughput via the multi-DATA train, multi-RTS reception, and BACK.

## 1. Introduction

In recent years, underwater communications and networking technologies for underwater wireless sensor networks (UWSNs) and the Internet of Underwater Things (IoUT) have been studied and developed actively by academia and industrial researchers due to their challenges and applications [1,2,3,4]. Felemban et al. [1] provided a comprehensive survey on UWSN applications including water quality monitoring, habitat monitoring, fish farming, natural resource explorations, oil and gas pipeline monitoring, disaster forecasting (such as volcanoes and earthquakes), military surveillance, mine detection, and assistive navigation. Kao et al. [2] presented a comprehensive study on IoUT applications, challenges for IoUT, and UWSN channel models. For the main challenges for IoUT, they discussed the differences between UWSNs and territorial (or terrestrial) wireless sensor networks (TWSNs) including transmission media, rate, range, and propagation speed. Jouhari et al. [3] provided a survey on localization protocols and enabling technologies for UWSNs and IoUT including magneto-inductive communications and acoustic communications. In [3], the magneto-inductive communications can be used for short range and high data rate while the acoustic communications can be used for long range and low data rate. Ali et al. [4] presented a survey of technical issues and future directions for electromagnetic, optical, and acoustic communications in underwater environments. They also presented related issues of UWSNs and emerging technologies in underwater environmental communications.

There have been many studies on medium access control (MAC) protocols in TWSNs. IEEE 802.11 standard [5] has presented the distributed coordination function (DCF) based on carrier sense multiple access with collision avoidance (CSMA/CA) for wireless local area networks (WLANs). Tinnirello et al. [6] proposed a modeling approach to analyze the throughput and delay performance of IEEE 802.11 DCF. In [7], an analytical model of IEEE 802.11 DCF with a back-off freezing mechanism was proposed for WLANs. Sthapit and Pyun [8] proposed an implicit block acknowledgment (ACK) scheme that does not use the explicit block ACK request frame for WLANs. In [9], a method to optimize the number of aggregated MAC protocol data units was proposed for maximizing the system throughput with considering delay requirements in IEEE 802.11ac. In IEEE 802.15.4 standard [10] for low-rate wireless networks, the MAC protocol based on the slotted CSMA/CA mechanism is used in a contention access period. In [11], a method to estimate the number of active devices was proposed for the slotted CSMA/CA without acknowledgment in IEEE 802.15.4.

To consider the differences between TWSNs and USWNs such as transmission media, rate, range, and propagation speed, there have been many studies on MAC protocols in USWNs. Chen et al. [12] provided a survey on MAC protocols including contention-free, contention-based, and hybrid MAC protocols for UWSNs. Jiang [13] provided a survey on MAC protocols for underwater acoustic networks based on a MAC reference model. A slotted floor acquisition multiple access (FAMA) was presented by Molins and Stojanovic [14]. Slotted FAMA uses time slotting, carrier sensing, and a handshake of request to send (RTS) and clear to send (CTS) control packets. Ng et al. [15] proposed the MACA-U protocol by adapting terrestrial multiple access collision avoidance (MACA) for underwater acoustic networks. Chirdchoo et al. [16] proposed MACA-MN protocol based on MACA with packet train for multiple neighbors in underwater acoustic networks. As our previous study [17], an underwater CSMA/CA protocol with multi-RTS and multi-DATA receptions was proposed and analyzed in terms of throughput and delay. For underwater acoustic networks, Anjangi and Chitre [18] formulated an optimization problem to minimize the fractional idle time in a frame for unslotted variable packet duration schedules. Kim et al. [19] proposed UCMAC protocol which is an underwater cooperative MAC protocol for UWSNs as a reactive type. Cho et al. [20] proposed an asymmetric propagation delay-aware time division multiple access protocol for mobile acoustic UWSNs requiring periodic transmission of data. Yang et al. [21] proposed a dual channel MAC protocol for acoustic UWSNs where each node uses two underwater acoustic antennas that one is directional and the other is omnidirectional. Wang and Zhao [22] proposed a handshake-competition MAC protocol with a power control for underwater acoustic networks. Zhou et al. [23] proposed a dynamic timeslot contention MAC protocol based on predicting positions of autonomous underwater vehicles (AUVs). Xi et al. [24] proposed an underwater multi-channel MAC protocol which is based on underwater localization and a single transceiver. A two-dimensional discrete Markov chain is presented to model the back-off mechanism. The underwater localization is based on the time difference of arrival (TDoA) that may not need time synchronization.

In USWNs, underwater localization can be an important issue and there have been many studies on underwater localization techniques. Su et al. [25] presented a review of localization techniques for acoustic UWSNs. Underwater localization techniques can belong to distributed localization or centralized localization. They can also fall into prediction-based localization or estimated localization. The received signal strength indicator, the time of arrival (ToA), TDoA, and the angle of arrival can be used for underwater localization techniques. Zheng et al. [26] proposed a localization approach for mobile nodes in acoustic UWSNs. The localization approach utilizes a Kalman filter to align the timestamps received from neighboring anchor nodes. Since the localization approach also utilizes a penalty convex-concave procedure and sound ray tracing, the lake experiment and the deep sea experiment show that the localization error for the mobile node can be reduced. Bo et al. [27] presented a method to optimize the formation of AUVs for three-dimensional cooperative localization of AUVs. Since the method utilizes the measurements of TDoA, the method can reduce the localization error by increasing the information related to TDoA. Luo et al. [28] proposed a mobility-assisted localization scheme that can be suitable for three-dimensional large-scale UWSNs. The localization scheme considers the measurement of distance between sensor nodes that does not need time synchronization. The localization scheme utilizes two-way ToA for the localization of the remaining sensor nodes.

For USWNs, there have been many studies on other issues including resource allocation and routing protocols. Song et al. [29] proposed resource allocation algorithms for the downlink of an acoustic UWSN that consists of underwater sensor nodes, buoy nodes, and a base station on a ship. They consider that the buoy node can act as a relay and harvest the energy. In the downlink of an acoustic UWSN, one resource allocation algorithm is presented to maximize the sum rate and another resource allocation algorithm is presented to maximize the energy efficiency. Coutinho et al. [30] proposed a geographic and opportunistic routing protocol for a UWSN that consists of underwater sensor nodes, surface sonobuoys, and a monitoring center. The routing protocol can route a data packet from an underwater sensor node to some surface sonobuoys using the anycast nature. It is assumed that if a surface sonobuoy receives a data packet, it can forward the data packet to a monitoring center. The routing protocol utilizes a periodic beaconing algorithm, a next-hop forwarder set selection algorithm, and a void node recovery algorithm. Since each underwater sensor node is assumed to know its own location, the location of neighbor nodes can be disseminated via the periodic beaconing algorithm. Jafri et al. [31] proposed an analytical model to study the optimal depth threshold of a receiver-based opportunistic routing protocol for a UWSN. The UWSN consists of underwater motes and surface sonobuoys. The receiver-based opportunistic routing protocol decides to forward a packet using the depth difference between a transmitter and a receiver. They presented a numerical method for computing the optimal depth threshold by assuming homogeneous Poisson point processes.

In this paper, we propose an underwater multi-DATA train protocol with multi-RTS reception and block ACK (BACK), shortly called an underwater multi-DATA train protocol, based on the CSMA/CA protocol using the RTS/CTS exchange method. We consider an underwater acoustic sensor network which consists of an underwater sink node and multiple underwater sensor nodes. Each underwater sensor node can transmit sensory information to the underwater sink node using our proposed protocol. Due to long underwater acoustic propagation delay, some RTS frames may not overlap at a sink node, even if the RTS frames were sent to the sink node simultaneously by different sensor nodes. We consider that our underwater sink node can recover these nonoverlapping RTS frames, that we can call multi-RTS reception. Since our RTS frame contains ID of the RTS sending node and a timestamp, the sink node calculates the propagation delay between the RTS sending node and the sink node, then broadcasts a CTS frame. Since our CTS frame contains the time information about when each RTS sending node can transmit a DATA frame to the sink node, multiple DATA frames transmitted from different sensor nodes to the sink node can be formed as a train, that we can call a multi-DATA train. Then, the sink node broadcasts a BACK frame which contains whether each DATA frame was successfully received or not. We also propose an underwater BACK protocol which is analogous to our proposed underwater multi-DATA train protocol except the multi-DATA train and the information contained in RTS and CTS frames. Then, we analyze normalized throughput and mean access delay of our proposed underwater multi-DATA train protocol, our proposed underwater BACK protocol, and the conventional protocols, respectively. The analytical results agree very well with the simulation results for throughput and delay performances of our proposed protocols and the conventional protocols, respectively, with various numbers of underwater sensor nodes and input parameters. The analytical results and the simulation results show that our proposed protocols outperform the conventional protocols. We also provide a more generalized and accurate analysis of the throughput and delay performance for the underwater multi-RTS reception protocol presented in our previous study [17]. The throughput and delay performance of the underwater multi-RTS reception protocol is compared with that of our proposed underwater multi-DATA train protocol and that of our proposed underwater BACK protocol, respectively, for various values of input parameters. The analytical results and the simulation results show that our proposed underwater multi-DATA train protocol and our proposed underwater BACK protocol outperform the underwater multi-RTS reception protocol in terms of normalized throughput and mean access delay.

## 2. An Underwater Multi-DATA Train Protocol with Multi-RTS Reception and BACK

**Figure 1:**
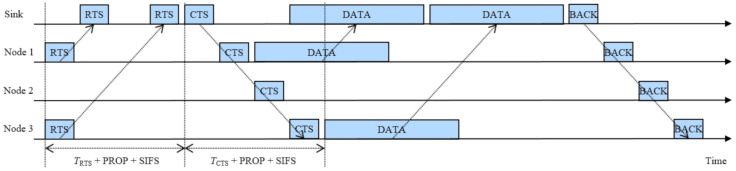
An example of the proposed underwater multi-DATA train protocol.

We consider an underwater acoustic sensor network which consists of an underwater sink node and multiple underwater sensor nodes. In the underwater acoustic sensor network, we consider that the underwater sink node and the underwater sensor nodes can hear each other via an underwater acoustic channel. Each underwater sensor node can transmit sensory information to the underwater sink node using our proposed underwater multi-DATA train protocol with multi-RTS reception and BACK, which can be shortly called an *underwater multi-DATA train protocol*. As shown in Figure 1, the proposed underwater multi-DATA train protocol is based on the CSMA/CA protocol using the RTS/CTS exchange method. An underwater sensor node, which has sensory information to transmit to an underwater sink node, can check the status of an underwater medium by performing the carrier sensing via an underwater acoustic channel. If the sensor node cannot hear any frame via the underwater acoustic channel during a slot time, the sensor node decrements the value of its own back-off counter by one. Otherwise, the sensor node freezes the value of its own back-off counter. If the value of back-off counter of the sensor node becomes zero, the sensor node sends an RTS frame to the underwater sink node. In the proposed underwater multi-DATA train protocol, our RTS frame contains ID of the RTS sending node and a timestamp. Using this timestamp, the underwater sink node can calculate the underwater propagation delay between the RTS sending node and the sink node. If more than one sensor node sends RTS frames simultaneously, the sent RTS frames may collide at the sink node. But, due to long underwater acoustic propagation delay, some sent RTS frames may not overlap at the sink node in the time domain, even if the RTS frames were sent from different sensor nodes to the sink node simultaneously. We consider that our underwater sink node can recover these nonoverlapping multiple RTS frames, that we can call *multi-RTS reception* at the underwater sink node. Using the recovered RTS frames each of which contains ID of its sending node and a timestamp, the underwater sink node calculates the underwater propagation delay between each RTS sending node and the sink node. Using the calculated propagation delay and ID of each RTS sending node, the underwater sink node broadcasts a CTS frame. In the proposed underwater multi-DATA train protocol, our CTS frame contains the time information about when each RTS sending node can transmit a DATA frame to the underwater sink node. From this time information contained in our CTS frame, multiple DATA frames each of which is transmitted from each RTS sending node to the sink node can be formed as a train, that we can call a *multi-DATA train* formed at the sink node. The time information in our CTS frame can make each RTS sending node transmit a DATA frame to the sink node in ascending order of the calculated propagation delay between each RTS sending node and the sink node. The time information in our CTS frame can also make the nearest RTS sending node to the sink node transmit a DATA frame immediately after receiving the CTS frame and a short guard time. The short guard time can be a short inter-frame space (SIFS) [5,6]. The multi-DATA train formed at the sink node can contain a short guard time between DATA frames. After receiving the train of multiple DATA frames from the RTS sending nodes, the underwater sink node broadcasts a BACK frame which contains the information about whether each DATA frame was successfully received or not. Each underwater sensor node which transmitted the DATA frame chooses a back-off counter value randomly between zero and a contention window size. The contention window size can be controlled to enhance the performance of the proposed underwater multi-DATA train protocol.

Figure 1 shows an example of the proposed underwater multi-DATA train protocol where $T_{\mathrm{RTS}}$ denotes the duration of RTS frame, $T_{\mathrm{CTS}}$ denotes the duration of CTS frame, and PROP denotes the underwater acoustic propagation delay between a sink node and the farthest underwater sensor node to the sink node in an underwater cell. For example, the sensor node 1 and the sensor node 3 send RTS frames simultaneously, then the RTS frames sent by the sensor nodes 1 and 3 may not overlap at the sink node due to long underwater acoustic propagation delay as shown in Figure 1.

In Figure 1, using the recovered RTS frames each of which contains ID of the sensor node 1 or 3 and a timestamp, the sink node calculates the underwater propagation delay between the sensor node 1 or 3 and the sink node. Then, the underwater sink node broadcasts a CTS frame which contains the time information about when the sensor nodes 1 and 3 can transmit a DATA frame to the underwater sink node, respectively. From this time information in our CTS frame, the DATA frames transmitted from the sensor nodes 1 and 3 to the sink node can be formed as a train at the sink node. The time information in our CTS frame can make the sensor nodes 1 and 3 transmit a DATA frame to the sink node in ascending order of the calculated propagation delay between each RTS sending node and the sink node. The time information in our CTS frame can also make the sensor node 1 which is the nearest RTS sending node to the sink node transmit a DATA frame immediately after receiving the CTS frame and a short guard time. The multi-DATA train formed at the sink node can contain a short guard time between the DATA frames. After receiving the multi-DATA train from the sensor nodes 1 and 3, the underwater sink node broadcasts a BACK frame which contains the information about whether each DATA frame was successfully received or not.

**Figure 2:**
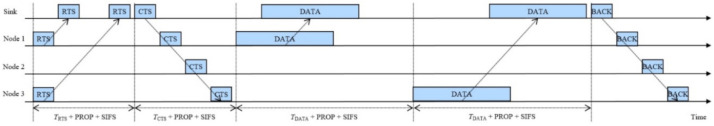
An example of the proposed underwater BACK protocol.

We also propose an underwater BACK protocol with multi-RTS reception, which can be shortly called an *underwater BACK protocol*. As shown in Figure 2, our proposed underwater BACK protocol is analogous to our proposed underwater multi-DATA train protocol except the multi-DATA train and the information contained in RTS and CTS frames. In the proposed underwater BACK protocol, an RTS frame does not need to contain a timestamp because an underwater sink node does not need to calculate the underwater propagation delay between the underwater sink node and each sensor node that sends the RTS frame. In the proposed underwater BACK protocol, a CTS frame does not need to contain the time information about when each sensor node that sent the recovered RTS frame can transmit a DATA frame to the underwater sink node. The CTS frame needs to contain the transmission order of DATA frames and ID of each sensor node that sent the recovered RTS frame. In this transmission order of DATA frames, each sensor node that sent the recovered RTS frame can transmit a DATA frame to the underwater sink node. After receiving DATA frames from the underwater sensor nodes, the underwater sink node broadcasts a BACK frame which contains the information about whether each DATA frame was successfully received or not.

Figure 2 shows an example of the proposed underwater BACK protocol where $T_{\mathrm{DATA}}$ denotes the duration of DATA frame. For example, the sensor node 1 and the sensor node 3 send RTS frames simultaneously, then the RTS frames sent by the sensor nodes 1 and 3 may not overlap at the sink node due to long underwater acoustic propagation delay as shown in Figure 2. Then, the underwater sink node broadcasts a CTS frame which contains the transmission order of DATA frames and IDs of the sensor nodes 1 and 3 each of which sent the recovered RTS frame. In this transmission order of DATA frames, the sensor nodes 1 and 3 can transmit a DATA frame to the underwater sink node, respectively. Since the CTS frame in the proposed underwater BACK protocol does not need to contain the time information about when each sensor node that sent the recovered RTS frame transmits a DATA frame to the underwater sink node, there can be a waste of time from when the sink node receives a DATA frame from the sensor node 1 to when the sink node receives a DATA frame from the sensor node 3. After receiving DATA frames from the sensor nodes 1 and 3, the sink node broadcasts a BACK frame which contains the information about whether each DATA frame was successfully received or not.

## 3. Successful Transmission Probabilities for the Proposed Underwater Multi-DATA Train Protocol

In this section, we calculate successful transmission probabilities for our proposed underwater multi-DATA train protocol. We consider that an underwater sink node is located at the center of an underwater cell and multiple underwater sensor nodes are distributed in the underwater cell. The underwater cell can be divided into r rings [17]. Each ring i=1,…,r has the boundary $\left[l_{i-1},l_{i}\right)$ where $l_{0}=0$ and the ring i has two radii $l_{i-1}$ and $l_{i}$. Let $d_{x,a}$ denote the distance between the underwater sensor node x in the ring a and the underwater sink node. Let $d_{y,b}$ denote the distance between the underwater sensor node y in the ring b and the underwater sink node. If the underwater sensor node x in the ring a and the underwater sensor node y in the ring b send RTS frames simultaneously to the underwater sink node and the following condition (1) is satisfied, the RTS frames sent by the underwater sensor nodes x and y to the underwater sink node may not overlap each other at the underwater sink node in the time domain as:

(1) $$\left|d_{x,a}-d_{y,b}\right|\geq v_{\mathrm{UWA}}\cdot \left(T_{\mathrm{RTS}}+\mathrm{SIFS}\right),$$

where $v_{\mathrm{UWA}}$ denotes the speed of sound via the underwater acoustic channel, $T_{\mathrm{RTS}}$ denotes the duration of RTS frame, and SIFS denotes a short inter-frame space [5,6]. When a=b, as the probability that RTS frames sent simultaneously from the same ring to a sink node overlap each other at the sink node is high, the RTS frames are assumed to be overlapped at the sink node. When a≠b, as the probability that RTS frames sent simultaneously from different rings to a sink node overlap each other at the sink node is low, the RTS frames are assumed to be not overlapped at the sink node. Let $N_{r}$ denote the number of underwater sensor nodes in an underwater cell when the number of rings is r. Let $n_{i}$ denote the number of underwater sensor nodes located in the ring i=1,…,r. The number of underwater sensor nodes $N_{r}$ can be expressed as:

(2) $$N_{r}=\overset{r}{\underset{i=1}{\sum }}n_{i}.$$

If at least one of underwater sensor nodes among $N_{r}$ sensor nodes sends an RTS frame, the underwater acoustic channel becomes busy. The channel busy probability in the case of r rings $p_{B|r}$ can be obtained as:

(3) $$p_{B|r}=1-{\left(1-p_{T|r}\right)}^{N_{r}},$$

where $p_{T|r}$ is the probability that an underwater sensor node sends an RTS frame to an underwater sink node when the number of rings is r.

When the number of rings r=1, let $p_{S|r=1}$ denote the probability that an RTS frame is successfully transmitted from an underwater sensor node to an underwater sink node. If the RTS frame is transmitted from only one underwater sensor node among $N_{1}$ sensor nodes, the RTS frame is successfully transmitted to the underwater sink node as:

(4) $$p_{B|r=1}\cdot p_{S|r=1}=N_{1}p_{T|r=1}{\left(1-p_{T|r=1}\right)}^{N_{1}-1}.$$

When the number of rings r=2, let $p_{S,\langle 1,2\rangle |r=2}$ denote the probability that two RTS frames are successfully and simultaneously transmitted from two underwater sensor nodes in two rings to an underwater sink node. If each of two RTS frames is transmitted from only one sensor node among $n_{1}$ sensor nodes in the ring 1 and only one sensor node among $n_{2}$ sensor nodes in the ring 2, respectively, the two RTS frames are successfully and simultaneously transmitted to the underwater sink node as:

(5) $$p_{B|r=2}\cdot p_{S,\langle 1,2\rangle |r=2}=n_{1}n_{2}p_{T|r=2}{}^{2}{\left(1-p_{T|r=2}\right)}^{N_{2}-2}.$$

Let $p_{S,\langle i\rangle |r=2}$ for i=1, 2 denote the probability that an RTS frame is successfully transmitted from an underwater sensor node in the ring i to an underwater sink node and RTS frames are not successfully transmitted from underwater sensor nodes in the other ring between two rings to the underwater sink node. If an RTS frame is transmitted from only one underwater sensor node among $n_{i}$ sensor nodes in the ring i and no or more than one RTS frame is transmitted from underwater sensor nodes among $N_{2}-n_{i}$ sensor nodes in the other ring, only one RTS frame is successfully transmitted from the sensor node in the ring i to the underwater sink node with the following probability $p_{S,\langle i\rangle |r=2}$ for i=1, 2 as:

(6) $$p_{B|r=2}\cdot p_{S,\langle 1\rangle |r=2}=n_{1}\overset{n_{2}}{\underset{j=0,j\neq 1}{\sum }}\left(\begin{array}{c} n_{2}\\ j \end{array}\right)p_{T|r=2}{}^{1+j}{\left(1-p_{T|r=2}\right)}^{N_{2}-1-j}.$$

(7) $$p_{B|r=2}\cdot p_{S,\langle 2\rangle |r=2}=n_{2}\overset{n_{1}}{\underset{j=0,j\neq 1}{\sum }}\left(\begin{array}{c} n_{1}\\ j \end{array}\right)p_{T|r=2}{}^{1+j}{\left(1-p_{T|r=2}\right)}^{N_{2}-1-j}.$$

Let $p_{S,m|r}$ denote the probability that the number of RTS frames transmitted successfully and simultaneously from underwater sensor nodes to an underwater sink node is m when the number of rings is r. The probability $p_{S|r=2}$ that one or more RTS frames are successfully and simultaneously transmitted from underwater sensor nodes to an underwater sink node when r=2 can be obtained as:

(8) $$p_{S|r=2}=\overset{2}{\underset{m=1}{\sum }}p_{S,m|r=2},$$

where the probabilities $p_{S,2|r=2}$ and $p_{S,1|r=2}$ can be derived as:

(9) $$p_{S,2|r=2}=p_{S,\langle 1,2\rangle |r=2},$$

(10) $$p_{S,1|r=2}=p_{S,\langle 1\rangle |r=2}+p_{S,\langle 2\rangle |r=2}.$$

When the number of rings r=3, let $p_{S,\langle 1,2,3\rangle |r=3}$ denote the probability that three RTS frames are successfully and simultaneously transmitted from three underwater sensor nodes in three rings to an underwater sink node. If each of three RTS frames is transmitted from only one sensor node among $n_{1}$ sensor nodes in the ring 1, only one sensor node among $n_{2}$ sensor nodes in the ring 2, and only one sensor node among $n_{3}$ sensor nodes in the ring 3, respectively, the three RTS frames are successfully and simultaneously transmitted to the underwater sink node as:

(11) $$p_{B|r=3}\cdot p_{S,\langle 1,2,3\rangle |r=3}=n_{1}n_{2}n_{3}p_{T|r=3}{}^{3}{\left(1-p_{T|r=3}\right)}^{N_{3}-3},$$

Let $p_{S,\langle i,j\rangle |r=3}$ for 〈i,j〉=〈1,2〉,  〈1,3〉,  〈2,3〉 denote the probability that two RTS frames are successfully and simultaneously transmitted from one sensor node in the ring i and one sensor node in the ring j to an underwater sink node and RTS frames are not successfully transmitted from underwater sensor nodes in the other ring to the underwater sink node. If an RTS frame is transmitted from only one sensor node among $n_{i}$ sensor nodes in the ring i, an RTS frame is transmitted from only one sensor node among $n_{j}$ sensor nodes in the ring j, and no or more than one RTS frame is transmitted from underwater sensor nodes among $N_{3}-n_{i}-n_{j}$ sensor nodes in the other ring, the two RTS frames are successfully and simultaneously transmitted from the sensor node in the ring i and the sensor node in the ring j to the underwater sink node with the following probability $p_{S,\langle i,j\rangle |r=3}$ for 〈i,j〉=〈1,2〉,  〈1,3〉,  〈2,3〉 as:

(12) $$p_{B|r=3}\cdot p_{S,\langle 1,2\rangle |r=3}=n_{1}n_{2}\overset{n_{3}}{\underset{k=0,k\neq 1}{\sum }}\left(\begin{array}{c} n_{3}\\ k \end{array}\right)p_{T|r=3}{}^{2+k}{\left(1-p_{T|r=3}\right)}^{N_{3}-2-k},$$

(13) $$p_{B|r=3}\cdot p_{S,\langle 1,3\rangle |r=3}=n_{1}n_{3}\overset{n_{2}}{\underset{k=0,k\neq 1}{\sum }}\left(\begin{array}{c} n_{2}\\ k \end{array}\right)p_{T|r=3}{}^{2+k}{\left(1-p_{T|r=3}\right)}^{N_{3}-2-k},$$

(14) $$p_{B|r=3}\cdot p_{S,\langle 2,3\rangle |r=3}=n_{2}n_{3}\overset{n_{1}}{\underset{k=0,k\neq 1}{\sum }}\left(\begin{array}{c} n_{1}\\ k \end{array}\right)p_{T|r=3}{}^{2+k}{\left(1-p_{T|r=3}\right)}^{N_{3}-2-k}.$$

Let $p_{S,\langle i\rangle |r=3}$ for i=1, 2, 3 denote the probability that an RTS frame is successfully transmitted from an underwater sensor node in the ring i to an underwater sink node and RTS frames are not successfully transmitted from underwater sensor nodes in the other rings to the underwater sink node. If an RTS frame is transmitted from only one underwater sensor node among $n_{i}$ sensor nodes in the ring i and no or more than one RTS frame is transmitted from underwater sensor nodes in each of the other rings, only one RTS frame is successfully transmitted from the sensor node in the ring i to the underwater sink node with the following probability $p_{S,\langle i\rangle |r=3}$ for i=1, 2, 3 as:

(15) $$p_{B|r=3}\cdot p_{S,\langle 1\rangle |r=3}=n_{1}\overset{n_{2}}{\underset{j=0,j\neq 1}{\sum }}\overset{n_{3}}{\underset{k=0,k\neq 1}{\sum }}\left(\begin{array}{c} n_{2}\\ j \end{array}\right)\left(\begin{array}{c} n_{3}\\ k \end{array}\right)p_{T|r=3}{}^{1+j+k}{\left(1-p_{T|r=3}\right)}^{N_{3}-1-j-k},$$

(16) $$p_{B|r=3}\cdot p_{S,\langle 2\rangle |r=3}=n_{2}\overset{n_{1}}{\underset{j=0,j\neq 1}{\sum }}\overset{n_{3}}{\underset{k=0,k\neq 1}{\sum }}\left(\begin{array}{c} n_{1}\\ j \end{array}\right)\left(\begin{array}{c} n_{3}\\ k \end{array}\right)p_{T|r=3}{}^{1+j+k}{\left(1-p_{T|r=3}\right)}^{N_{3}-1-j-k},$$

(17) $$p_{B|r=3}\cdot p_{S,\langle 3\rangle |r=3}=n_{3}\overset{n_{1}}{\underset{j=0,j\neq 1}{\sum }}\overset{n_{2}}{\underset{k=0,k\neq 1}{\sum }}\left(\begin{array}{c} n_{1}\\ j \end{array}\right)\left(\begin{array}{c} n_{2}\\ k \end{array}\right)p_{T|r=3}{}^{1+j+k}{\left(1-p_{T|r=3}\right)}^{N_{3}-1-j-k}.$$

The probability $p_{S|r=3}$ that one or more RTS frames are successfully and simultaneously transmitted from underwater sensor nodes to an underwater sink node when r=3 can be obtained as:

(18) $$p_{S|r=3}=\overset{3}{\underset{m=1}{\sum }}p_{S,m|r=3},$$

where the probabilities $p_{S,m|r=3}$ for m=1, 2, 3 can be derived as:

(19) $$p_{S,3|r=3}=p_{S,\langle 1,2,3\rangle |r=3},$$

(20) $$p_{S,2|r=3}=p_{S,\langle 1,2\rangle |r=3}+p_{S,\langle 1,3\rangle |r=3}+p_{S,\langle 2,3\rangle |r=3},$$

(21) $$p_{S,1|r=3}=p_{S,\langle 1\rangle |r=3}+p_{S,\langle 2\rangle |r=3}+p_{S,\langle 3\rangle |r=3}.$$

## 4. Analysis of Throughput and Delay for the Underwater Protocols

In this section, in order to compare the performances of our proposed protocols in the case of r rings with the performances of other protocols, we analyze the throughput performance $S_{protocol|r}$ and the delay performance $D_{protocol|r}$ of our proposed underwater multi-DATA train protocol ($S_{\mathrm{train}|r}$ and $D_{\mathrm{train}|r}$), our proposed underwater BACK protocol ($S_{\mathrm{BACK}|r}$ and $D_{\mathrm{BACK}|r}$), the underwater multi-RTS reception protocol ($S_{\mathrm{MRTS}|r}$ and $D_{\mathrm{MRTS}|r}$) [17], and the conventional CSMA/CA protocol ($S_{\mathrm{CONV}|r}$ and $D_{\mathrm{CONV}|r}$) based on [6,14]. The throughput performance $S_{protocol|r}$ is the normalized throughput defined as the time fraction of successful transmissions of DATA payloads over an underwater acoustic channel when the number of rings is r [6,17]. The normalized throughput $S_{protocol|r}$ for protocol∈ {CONV, MRTS, BACK, train} can be obtained as:

(22) $$S_{protocol|r}=\frac{p_{B|r}\cdot \sum_{m=1}^{r}\left(m\cdot p_{S,m|r}\right)\cdot T_{P}}{\left(1-p_{B|r}\right)\cdot T_{I}+p_{B|r}\cdot \left(1-p_{S|r}\right)\cdot T_{C}+p_{B|r}\cdot E\left[T_{protocol,S|r}\right]},\;\mathrm{for}\;protocol\in \left\{\mathrm{MRTS},\;\mathrm{BACK},\;\mathrm{train}\right\},$$

(23) $$S_{\mathrm{CONV}|r}=S_{\mathrm{MRTS}|r=1},$$

where $T_{P}$ denotes the duration of DATA payload, $T_{I}$ denotes the duration of an idle time slot, $E\left[T_{protocol,S|r}\right]$ for protocol∈ {MRTS, BACK, train} denotes the expected duration of successful transmissions of RTS, CTS, DATA, and ACK or BACK frames when the number of rings is r, and $T_{C}$ denotes the duration of colliding RTS transmissions. $T_{C}$ is considered as $T_{\mathrm{RTS}}+{\mathrm{PROP}}_{\max}+\mathrm{EIFS}$ where ${\mathrm{PROP}}_{\max}$ denotes the maximum underwater acoustic propagation delay between the farthest underwater sensor nodes in an underwater cell and EIFS denotes an extended inter-frame space [5,6]. The normalized throughput of the conventional CSMA/CA protocol $S_{\mathrm{CONV}|r}$ can be obtained as that of the underwater multi-RTS reception protocol $S_{\mathrm{MRTS}|r}$ in the case of r=1. The probabilities $p_{B|r}$, $p_{S|r}$, and $p_{S,m|r}$ in Equation (22) can be obtained from Equations (2)–(21). The expected duration $E\left[T_{protocol,S|r}\right]$ for protocol∈ {MRTS, BACK, train} in Equation (22) can be obtained from the following Equations (24)–(34).

When the underwater multi-RTS reception protocol with *r* rings is utilized, $E\left[T_{\mathrm{MRTS},S|r}\right]$ can be obtained as [17]:

(24) $$E\left[T_{\mathrm{MRTS},S|r}\right]=\overset{r}{\underset{m=1}{\sum }}p_{S,m|r}T_{\mathrm{MRTS},S,m},$$

(25) $$\begin{array}{c} T_{\mathrm{MRTS},S,m}=\left(T_{\mathrm{RTS}}+{\mathrm{PROP}}_{\max}+\mathrm{SIFS}\right)+\left(T_{\mathrm{CTS}}+{\mathrm{PROP}}_{\max}+\mathrm{SIFS}\right)+m\\ \cdot \left(T_{\mathrm{PHY}}+T_{\mathrm{MAC}}+T_{P}+{\mathrm{PROP}}_{\max}+\mathrm{SIFS}\right)+\left(m-1\right)\\ \cdot \left(T_{\mathrm{ACK}}+{\mathrm{PROP}}_{\max}+\mathrm{SIFS}\right)+T_{\mathrm{ACK}}+\mathrm{DIFS}, \end{array}$$

where $T_{\mathrm{CTS}}$ denotes the duration of CTS frame, $T_{\mathrm{PHY}}$ denotes the duration of PHY header, $T_{\mathrm{MAC}}$ denotes the duration of MAC header, $T_{\mathrm{ACK}}$ denotes the duration of ACK frame, and DIFS denotes a distributed inter-frame space [5,6].

When the proposed underwater BACK protocol with *r* rings is utilized, $E\left[T_{\mathrm{BACK},S|r}\right]$ can be obtained as:

(26) $$E[T_{\mathrm{BACK},S|r}]=\overset{r}{\underset{m=1}{\sum }}p_{S,m|r}T_{\mathrm{BACK},S,m},$$

(27) $$T_{\mathrm{BACK},S,m}=\left(T_{\mathrm{RTS}}+\mathrm{PROP}+\mathrm{SIFS}\right)+\left(T_{\mathrm{CTS}}+\mathrm{PROP}+\mathrm{SIFS}\right)+m\cdot \left(T_{\mathrm{PHY}}+T_{\mathrm{MAC}}+T_{P}\;+\mathrm{PROP}+\mathrm{SIFS}\right)+T_{\mathrm{BACK}}+\mathrm{DIFS},$$

where $T_{\mathrm{BACK}}$ denotes the duration of BACK frame and PROP denotes the underwater acoustic propagation delay between a sink node and the farthest underwater sensor node to the sink node in an underwater cell. Since the underwater sink node is considered to be located at the center of an underwater cell, PROP is considered as ${\mathrm{PROP}}_{\max}/2$.

When the proposed underwater multi-DATA train protocol with *r* rings is utilized, $E\left[T_{\mathrm{train},S|r}\right]$ can be obtained as:

(28) $$E[T_{\mathrm{train},S|r}]=\underset{\forall l|r}{\sum }p_{S,l|r}T_{\mathrm{train},S,l|r},$$

where l|r denotes an ordered list (tuple) of ring indexes when the number of rings is r, $p_{S,l|r}$ denotes the probability of the event that RTS frames are successfully and simultaneously transmitted from underwater sensor nodes in the rings whose indexes constitute the tuple l|r to an underwater sink node, and $T_{\mathrm{train},S,l|r}$ denotes the duration of successful transmissions of RTS, CTS, DATA, and BACK frames when the event of successful and simultaneous transmissions of RTS frames in the case of the tuple l|r occurs with the probability $p_{S,l|r}$ in the proposed underwater multi-DATA train protocol with *r* rings. When the number of rings r=2, the duration $T_{\mathrm{train},S,l|r}$ for the tuple $l\in \left\{\langle 1\rangle ,\langle 2\rangle ,\langle 1,2\rangle \right\}$ can be expressed as:

(29) $$T_{\mathrm{train},S,\langle i\rangle |r=2}=T_{\mathrm{BACK},S,1}-2\cdot \mathrm{PROP}+2\cdot {\mathrm{PROP}}_{i|r=2},\;\;\mathrm{for}\;i=1,\;2,$$

(30) $$T_{\mathrm{train},S,\langle 1,2\rangle |r=2}=T_{\mathrm{BACK},S,2}-3\cdot \mathrm{PROP}+2\cdot {\mathrm{PROP}}_{1|r=2},$$

where ${\mathrm{PROP}}_{i|r}$ denotes the underwater propagation delay between a sink node and the farthest underwater sensor node in the ring i to the sink node in an underwater cell when the number of rings is r, and ${\mathrm{PROP}}_{r|r}=\mathrm{PROP}$. If $\left|l_{2}-l_{1}\right|=\left|l_{1}-l_{0}\right|$ where the ring i has two radii $l_{i-1}$ and $l_{i}$, ${\mathrm{PROP}}_{1|r=2}=\mathrm{PROP}/2$. When the number of rings r=3, the duration $T_{\mathrm{train},S,l|r}$ for the tuple $l\in \left\{\langle 1\rangle ,\langle 2\rangle ,\langle 3\rangle ,\langle 1,2\rangle ,\langle 1,3\rangle ,\langle 2,3\rangle ,\langle 1,2,3\rangle \right\}$ can be expressed as:

(31) $$T_{\mathrm{train},S,\langle i\rangle |r=3}=T_{\mathrm{BACK},S,1}-2\cdot \mathrm{PROP}+2\cdot {\mathrm{PROP}}_{i|r=3},\;\;\mathrm{for}\;i=1,\;2,\;3,$$

(32) $$T_{\mathrm{train},S,\langle 1,2\rangle |r=3}=T_{\mathrm{train},S,\langle 1,3\rangle |r=3}=T_{\mathrm{BACK},S,2}-3\cdot \mathrm{PROP}+2\cdot {\mathrm{PROP}}_{1|r=3},$$

(33) $$T_{\mathrm{train},S,\langle 2,3\rangle |r=3}=T_{\mathrm{BACK},S,2}-3\cdot \mathrm{PROP}+2\cdot {\mathrm{PROP}}_{2|r=3},$$

(34) $$T_{\mathrm{train},S,\langle 1,2,3\rangle |r=3}=T_{\mathrm{BACK},S,3}-4\cdot \mathrm{PROP}+2\cdot {\mathrm{PROP}}_{1|r=3},$$

where ${\mathrm{PROP}}_{i|r=3}$ denotes ${\mathrm{PROP}}_{i|r}$ when the number of rings r=3, and ${\mathrm{PROP}}_{r|r}=\mathrm{PROP}$. If $\left|l_{3}-l_{2}\right|=\left|l_{2}-l_{1}\right|=\left|l_{1}-l_{0}\right|$, ${\mathrm{PROP}}_{i|r=3}=i\cdot {\mathrm{PROP}}_{1|r=3}$ for i=1, 2, 3. The duration $T_{\mathrm{train},S,l|r}$ is shorter than or equal to the duration $T_{\mathrm{BACK},S,m}$ when m is equal to the number of elements of the tuple l|r. It is because the underwater propagation delay between an underwater sink node and an underwater sensor node can be calculated and utilized in the proposed underwater multi-DATA train protocol. Thus, multiple DATA frames transmitted from underwater sensor nodes can be formed as a train at the underwater sink node in the proposed underwater multi-DATA train protocol.

The delay performance $D_{protocol|r}$ for protocol∈ {CONV, MRTS, BACK, train} is defined as the mean access delay from when an underwater sensor node has a DATA frame at the head of its own queue to when the underwater sensor node completes the reception of the acknowledgement for the DATA frame in the case of r rings [6,17]. The mean access delay $D_{protocol|r}$ for protocol∈ {CONV, MRTS, BACK, train} can be obtained as:

(35) $$D_{protocol|r}=\frac{N_{r}\cdot \left\{\left(1-p_{B|r}\right)\cdot T_{I}+p_{B|r}\cdot \left(1-p_{S|r}\right)\cdot T_{C}+p_{B|r}\cdot E[T_{protocol,S|r}]\right\}}{p_{B|r}\cdot \sum_{m=1}^{r}\left(m\cdot p_{S,m|r}\right)},\;\;\;\mathrm{for}\;protocol\in \left\{\mathrm{MRTS},\mathrm{BACK},\mathrm{train}\right\},$$

(36) $$D_{\mathrm{CONV}|r}=D_{\mathrm{MRTS}|r=1},$$

where the mean access delay of the conventional CSMA/CA protocol $D_{\mathrm{CONV}|r}$ can be obtained as that of the underwater multi-RTS reception protocol $D_{\mathrm{MRTS}|r}$ in the case of r=1. The probabilities $p_{B|r}$, $p_{S|r}$, and $p_{S,m|r}$ in Equation (35) can be obtained from Equations (2)–(21). The expected duration $E\left[T_{protocol,S|r}\right]$ for $protocol\in \left\{\mathrm{MRTS},\;\mathrm{BACK},\;\mathrm{train}\right\}$ in Equation (35) can be obtained from Equations (24)–(34).

## 5. Results and Discussion

In this section, we compare and discuss analytical results and simulation results of the normalized throughput $S_{protocol|r}$ and the mean access delay $D_{protocol|r}$ for protocol∈ {CONV, MRTS, BACK, train} in the case of r rings with various values of input parameters. For the analytical results and the simulation results, we consider the number of underwater sensor nodes as $N_{1}=N_{2}=N_{3}$ and $n_{i}=N_{r}/r$, for i=1,…,r when the number of rings is r. Each ring i=1,…,r has the boundary $\left[l_{i-1},l_{i}\right)$ where $l_{0}=0$ and the ring i has two radii $l_{i-1}$ and $l_{i}$. We consider $l_{i}=i\cdot l_{1}$ for i=1,…,r. We set $l_{r}=r_{C}$ where $r_{C}$ is a cell radius. We consider the transmission probability $p_{T|r}$ as $2/\left(W_{r}+2\right)$ where $W_{r}$ denotes a contention window size when the number of rings is r. We consider ${\mathrm{PROP}}_{i|r}=$ $i\cdot {\mathrm{PROP}}_{1|r}$ for i=1,…,r when the number of rings is r. In our simulation, the condition (1) for two underwater sensor nodes is satisfied when the two underwater sensor nodes are located in different rings. In our simulation, the condition (1) for two underwater sensor nodes is not satisfied when the two underwater sensor nodes are located in the same ring. The input parameters and values for the analytical and simulation results are shown in Table 1 based on [2,3,4,5,6,17].

**Figure 3:**
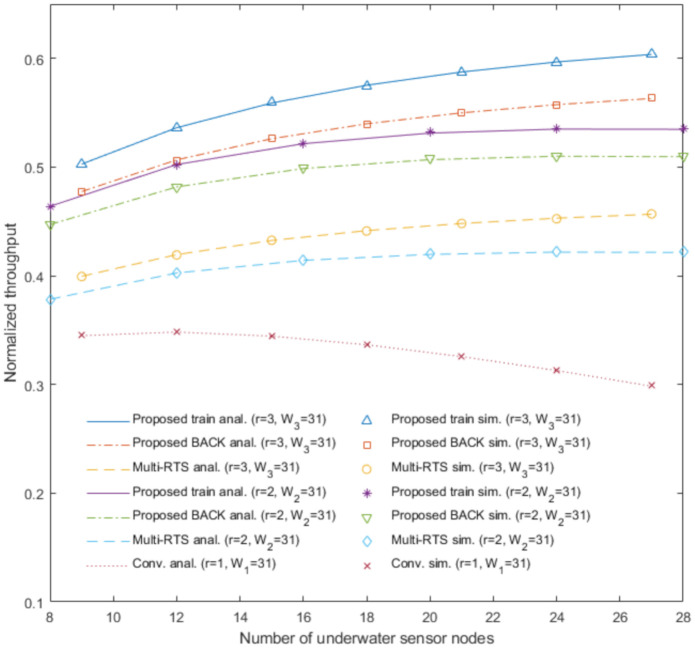
Normalized throughput of the proposed underwater multi-DATA train protocol, the proposed underwater BACK protocol, and the underwater multi-RTS reception protocol with *r* = 2 and *r* = 3, and the conventional CSMA/CA protocol with *r* = 1.

**Figure 4:**
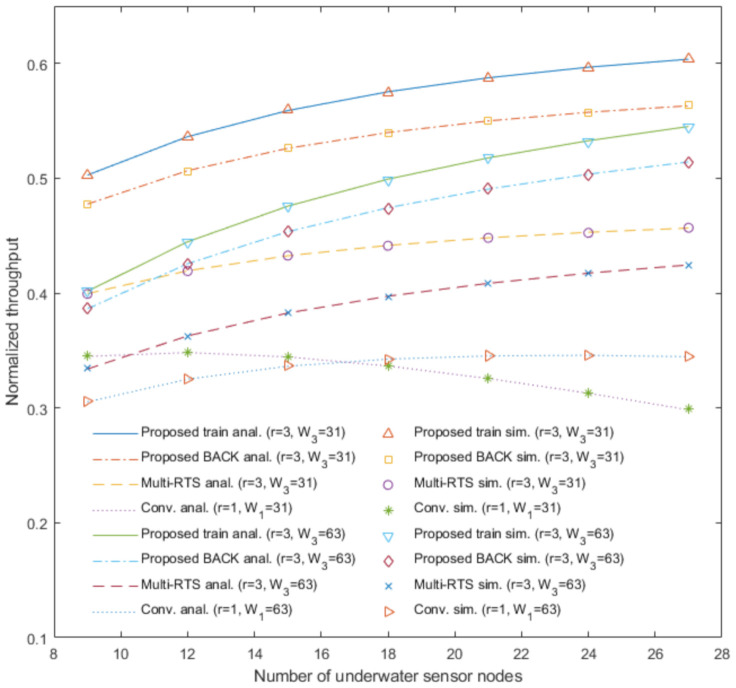
Normalized throughput of the proposed underwater multi-DATA train protocol, the proposed underwater BACK protocol, and the underwater multi-RTS reception protocol with $W_{3}$ = 31 and $W_{3}$ = 63, and the conventional CSMA/CA protocol with $W_{1}$ = 31 and $W_{1}$ = 63.

**Figure 5:**
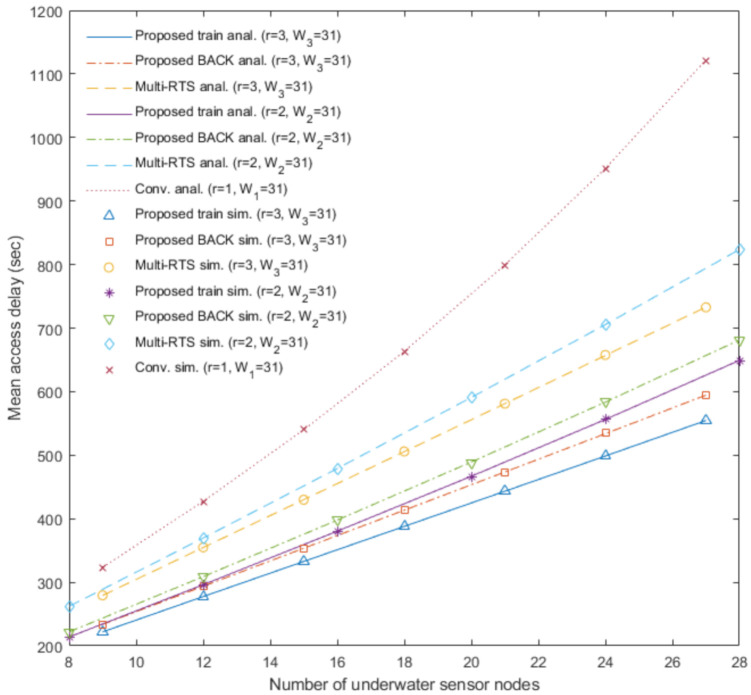
Mean access delay of the proposed underwater multi-DATA train protocol, the proposed underwater BACK protocol, and the underwater multi-RTS reception protocol with *r* = 2 and *r* = 3, and the conventional CSMA/CA protocol with *r* = 1.

**Figure 6:**
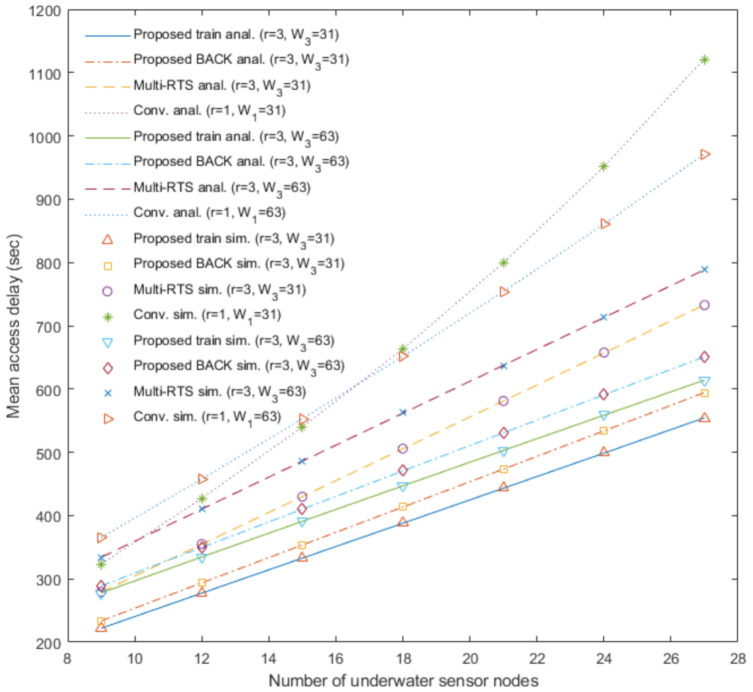
Mean access delay of the proposed underwater multi-DATA train protocol, the proposed underwater BACK protocol, and the underwater multi-RTS reception protocol with $W_{3}$ = 31 and $W_{3}$ = 63, and the conventional CSMA/CA protocol with $W_{1}$ = 31 and $W_{1}$ = 63.

Figure 3, Figure 4, Figure 5 and Figure 6 show the analytical results (lines) and the simulation results (marks) of our proposed underwater multi-DATA train protocol, our proposed underwater BACK protocol, the underwater multi-RTS reception protocol [17], and the conventional CSMA/CA protocol based on [6,14], respectively, in terms of the normalized throughput and the mean access delay with varying the number of underwater sensor nodes $N_{r}$ when the number of rings is r.

Figure 3, Figure 4, Figure 5 and Figure 6 show that the analytical results of $S_{protocol|r}$ and $D_{protocol|r}$ for protocol∈ {CONV, MRTS, BACK, train} agree very well with the simulation results, respectively, for various values of $N_{r}$ and input parameters. Figure 3 shows the analytical results and the simulation results of the proposed underwater multi-DATA train protocol with *r* = 2 and *r* = 3, the proposed underwater BACK protocol with *r* = 2 and *r* = 3, the underwater multi-RTS reception protocol with *r* = 2 and *r* = 3, and the conventional CSMA/CA protocol with *r* = 1 in terms of the normalized throughput with varying the number of underwater sensor nodes $N_{r}$ when the contention window size $W_{r}$ = 31. Figure 3 shows that the proposed underwater BACK protocol with *r* = 2 and *r* = 3 outperforms the underwater multi-RTS reception protocol with *r* = 2 and *r* = 3, respectively, in terms of the normalized throughput. It is because the proposed underwater BACK protocol can save more time to acknowledge the reception of multiple DATA frames than the underwater multi-RTS reception protocol. Figure 3 also shows that the proposed underwater multi-DATA train protocol with *r* = 2 and *r* = 3 outperforms the proposed underwater BACK protocol with *r* = 2 and *r* = 3, respectively, in terms of the normalized throughput. It is because the proposed underwater multi-DATA train protocol can save more time to transmit multiple DATA frames than the proposed underwater BACK protocol. The analytical results and the simulation results show that the conventional CSMA/CA protocol with *r* = 1 may yield the lowest normalized throughput among these protocols. It is because the conventional CSMA/CA protocol with *r* = 1 cannot provide the multi-RTS reception capability for an underwater sink node. The analytical and simulation results also show that larger number of rings *r* can yield larger normalized throughput for the proposed underwater multi-DATA train protocol, the proposed underwater BACK protocol, and the underwater multi-RTS reception protocol, respectively. It is because an underwater sink node utilizing these protocols with more rings can recover more RTS frames transmitted simultaneously from underwater sensor nodes which are not overlapped at the underwater sink node due to long underwater acoustic propagation delay.

Figure 4 shows the analytical results and the simulation results of the proposed underwater multi-DATA train protocol with *r* = 3, the proposed underwater BACK protocol with *r* = 3, the underwater multi-RTS reception protocol with *r* = 3, and the conventional CSMA/CA protocol with *r* = 1 in terms of the normalized throughput with varying the number of underwater sensor nodes $N_{r}$ and the contention window size $W_{r}$. The analytical and simulation results show that the normalized throughput of the conventional CSMA/CA protocol with $W_{1}$ = 31 is larger than that with $W_{1}$ = 63 when the number of underwater sensor nodes is 9, 12, and 15. It is because underwater sensor nodes using the conventional CSMA/CA protocol with $W_{1}$ = 63 can wait more time to access the underwater medium than those with $W_{1}$ = 31 when the number of underwater sensor nodes is small. The analytical and simulation results show that the normalized throughput of the conventional CSMA/CA protocol with $W_{1}$ = 31 is smaller than that with $W_{1}$ = 63 when the number of underwater sensor nodes is 18, 21, 24, and 27. It is because the conventional CSMA/CA protocol with $W_{1}$ = 31 can cause more collisions due to more RTS frames transmitted simultaneously from underwater sensor nodes than that with $W_{1}$ = 63 when the number of underwater sensor nodes is large. Figure 4 shows that throughput performances $S_{protocol|r=3}$ for protocol∈ {MRTS, BACK, train} with $W_{3}$ = 31 are higher than those with $W_{3}$ = 63, respectively. It is because underwater sensor nodes utilizing these protocols with $W_{3}$ = 31 can wait less time to access the underwater medium than those with $W_{3}$ = 63. Figure 3 and Figure 4 generally show the rank of normalized throughput as: the proposed underwater multi-DATA train protocol > the proposed underwater BACK protocol > the underwater multi-RTS reception protocol > the conventional CSMA/CA protocol.

Figure 5 shows the analytical results and the simulation results of the proposed underwater multi-DATA train protocol with *r* = 2 and *r* = 3, the proposed underwater BACK protocol with *r* = 2 and *r* = 3, the underwater multi-RTS reception protocol with *r* = 2 and *r* = 3, and the conventional CSMA/CA protocol with *r* = 1 in terms of the mean access delay with varying the number of underwater sensor nodes $N_{r}$ when the contention window size $W_{r}$ = 31. The analytical and simulation results show that the conventional CSMA/CA protocol with *r* = 1 may yield the worst delay performance among these protocols with varying the values of $N_{r}$. The analytical and simulation results show that delay performances of the proposed underwater BACK protocol with *r* = 2 and *r* = 3 are better than those of the underwater multi-RTS reception protocol with *r* = 2 and *r* = 3, respectively. Since the proposed underwater BACK protocol transmits the BACK frame instead of multiple ACK frames, the proposed underwater BACK protocol can shorten the delay to acknowledge the reception of multiple DATA frames. The analytical and simulation results show that delay performances of the proposed underwater multi-DATA train protocol with *r* = 2 and *r* = 3 are better than those of the proposed underwater BACK protocol with *r* = 2 and *r* = 3, respectively. Since an underwater sink node utilizing the proposed underwater multi-DATA train protocol can receive a train of multiple successive DATA frames which has a short guard time between the successive DATA frames, the proposed underwater multi-DATA train protocol can shorten the delay for the underwater sink node to receive multiple DATA frames. The analytical and simulation results also show that more rings can yield better delay performance for the underwater multi-RTS reception protocol, the proposed underwater BACK protocol, and the proposed underwater multi-DATA train protocol, respectively. It is because these protocols in the case of more rings can make an underwater sink node recover more RTS frames transmitted simultaneously from multiple underwater sensor nodes which are not overlapped at the underwater sink node due to long underwater acoustic propagation delay.

Figure 6 shows the analytical results and the simulation results of the proposed underwater multi-DATA train protocol with *r* = 3, the proposed underwater BACK protocol with *r* = 3, the underwater multi-RTS reception protocol with *r* = 3, and the conventional CSMA/CA protocol with *r* = 1 in terms of the mean access delay with varying the number of underwater sensor nodes $N_{r}$ and the contention window size $W_{r}$. When the number of underwater sensor nodes is 18, 21, 24, and 27, the results show that the delay performance of the conventional CSMA/CA protocol with $W_{1}$ = 63 is better than that with $W_{1}$ = 31. It is because the conventional CSMA/CA protocol with $W_{1}$ = 63 can cause less collisions of RTS frames transmitted from underwater sensor nodes than that with $W_{1}$ = 31 when the number of underwater sensor nodes is large. When the number of underwater sensor nodes is 9, 12, and 15, the results show that the delay performance of the conventional CSMA/CA protocol with $W_{1}$ = 31 is better than that with $W_{1}$ = 63. It is because underwater sensor nodes using the conventional CSMA/CA protocol with $W_{1}$ = 31 can wait less time to access the underwater medium than those with $W_{1}$ = 63 when the number of underwater sensor nodes is small. The analytical and simulation results show that delay performances $S_{protocol|r=3}$ for protocol∈ {MRTS, BACK, train} with $W_{3}$ = 31 are better than those with $W_{3}$ = 63, respectively. It is because underwater sensor nodes utilizing these protocols with $W_{3}$ = 31 can shorten average waiting time to access the underwater medium than those with $W_{3}$ = 63. Figure 5 and Figure 6 generally show the rank of mean access delay as: the conventional CSMA/CA protocol > the underwater multi-RTS reception protocol > the proposed underwater BACK protocol > the proposed underwater multi-DATA train protocol.

**Figure 7:**
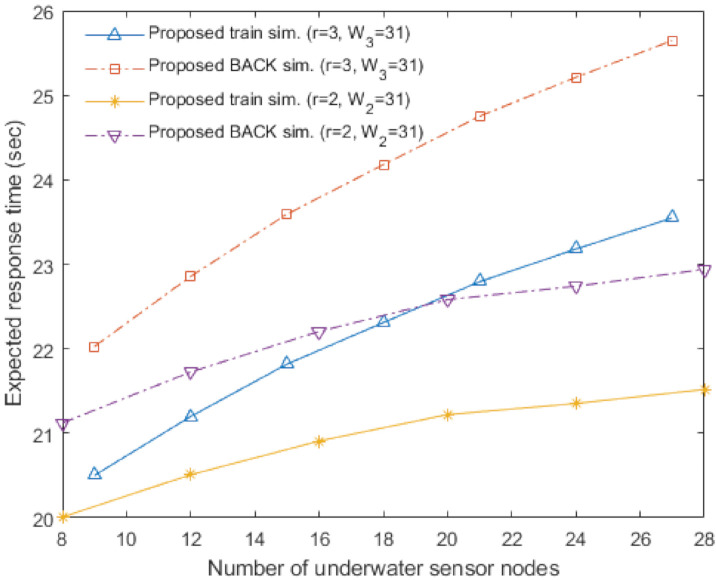
Expected response time of the proposed underwater multi-DATA train protocol with *r* = 2 and *r* = 3, and the proposed underwater BACK protocol with *r* = 2 and *r* = 3 when $W_{r}$ = 31.

Figure 7 shows the simulation results of the proposed underwater multi-DATA train protocol with *r* = 2 and *r* = 3, and the proposed underwater BACK protocol with *r* = 2 and *r* = 3 in terms of expected response time with varying the number of underwater sensor nodes $N_{r}$ when the contention window size $W_{r}$ = 31. In our simulation, we obtain the expected response time as an additional performance metric, where the expected response time is defined as the expected time from when an underwater sensor node transmits its RTS frame to when the underwater sensor node completes the reception of the acknowledgement for its DATA frame. The simulation results show that the expected response times of the proposed underwater multi-DATA train protocol with *r* = 2 and *r* = 3 are shorter than those of the proposed underwater BACK protocol with *r* = 2 and *r* = 3, respectively. It is because the proposed underwater multi-DATA train protocol can save more time to transmit multiple DATA frames than the proposed underwater BACK protocol. The simulation results also show that larger number of rings *r* can yield a longer expected response time for the proposed underwater multi-DATA train protocol and the proposed underwater BACK protocol, respectively. It is because an underwater sink node utilizing these protocols with more rings can recover more RTS frames transmitted simultaneously from underwater sensor nodes which are not overlapped at the underwater sink node due to long underwater acoustic propagation delay.

**Figure 8:**
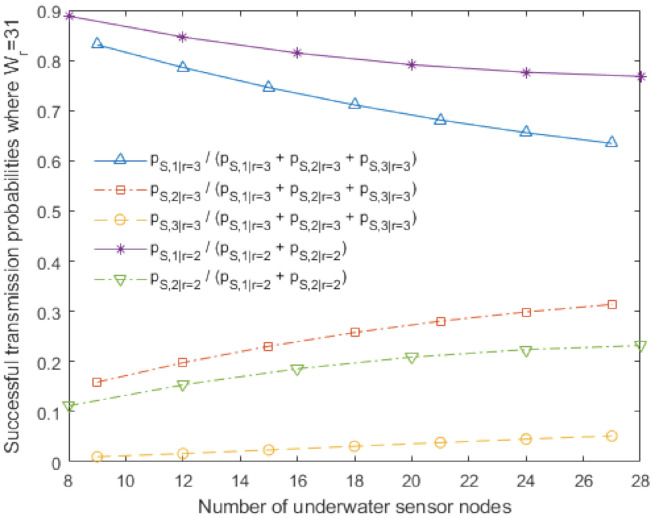
Successful transmission probabilities for the proposed underwater multi-DATA train protocol with *r* = 2 and *r* = 3 when $W_{r}$ = 31.

Figure 8 shows successful transmission probabilities for the proposed underwater multi-DATA train protocol with *r* = 2 and *r* = 3 for various numbers of underwater sensor nodes when the contention window size $W_{r}$ = 31. The analytical results in the case of *r* = 2 show $p_{S,1|r=2}/p_{S|r=2}$ and $p_{S,2|r=2}/p_{S|r=2}$ where $p_{S|r=2}=\sum_{m=1}^{2}p_{S,m|r=2}$. As the number of underwater sensor nodes increases, $p_{S,1|r=2}/p_{S|r=2}$ decreases and $p_{S,2|r=2}/p_{S|r=2}$ increases. It can yield that the expected response time of the proposed underwater multi-DATA train protocol with *r* = 2 and $W_{2}$ = 31 increases as the number of underwater sensor nodes increases as shown in Figure 7. The analytical results in the case of *r* = 3 show $p_{S,1|r=3}/p_{S|r=3}$, $p_{S,2|r=3}/p_{S|r=3}$, and $p_{S,3|r=3}/p_{S|r=3}$ where $p_{S|r=3}=\sum_{m=1}^{3}p_{S,m|r=3}$. As the number of underwater sensor nodes increases, $p_{S,1|r=3}/p_{S|r=3}$ decreases, $p_{S,2|r=3}/p_{S|r=3}$ increases, and $p_{S,3|r=3}/p_{S|r=3}$ increases. It can yield that the expected response time of the proposed underwater multi-DATA train protocol with *r* = 3 and $W_{3}$ = 31 increases as the number of underwater sensor nodes increases as shown in Figure 7.

**Figure 9:**
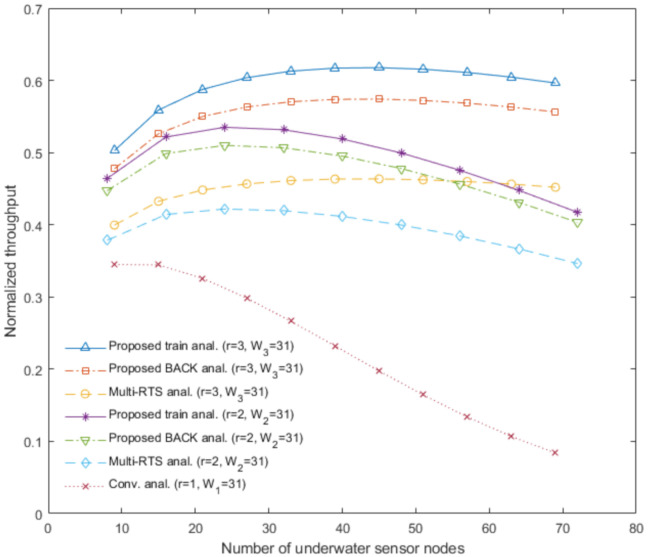
Normalized throughput versus the number of underwater sensor nodes for the proposed underwater multi-DATA train protocol, the proposed underwater BACK protocol, the underwater multi-RTS reception protocol, and the conventional CSMA/CA protocol.

Figure 9 shows the analytical results of the proposed underwater multi-DATA train protocol with *r* = 2 and *r* = 3, the proposed underwater BACK protocol with *r* = 2 and *r* = 3, the underwater multi-RTS reception protocol with *r* = 2 and *r* = 3, and the conventional CSMA/CA protocol with *r* = 1 in terms of the normalized throughput with varying the number of underwater sensor nodes $N_{r}$ when the contention window size $W_{r}$ = 31. Figure 9 shows the analytical results obtained with larger number of underwater sensor nodes $N_{r}$ for these protocols than Figure 3. 

In the case of *r* = 1 and $W_{1}$ = 31, Figure 9 shows that the conventional CSMA/CA protocol can achieve the highest normalized throughput when $N_{r}$ is about 9. In the case of *r* = 2 and $W_{2}$ = 31, Figure 9 shows that the proposed underwater multi-DATA train protocol, the proposed underwater BACK protocol, and the underwater multi-RTS reception protocol can achieve the highest normalized throughput when $N_{r}$ is about 24, respectively. In the case of *r* = 3 and $W_{3}$ = 31, Figure 9 shows that the proposed underwater multi-DATA train protocol, the proposed underwater BACK protocol, and the underwater multi-RTS reception protocol can achieve the highest normalized throughput when $N_{r}$ is about 45, respectively. Thus, the analytical results show that these protocols in the case of more rings can allow more underwater sensor nodes to transmit sensory information to an underwater sink node until the decay of throughput.

## 6. Conclusions

In this paper, we proposed an underwater multi-DATA train protocol with multi-RTS reception and BACK for underwater acoustic sensor networks. Due to long underwater acoustic propagation delay, some RTS frames may not overlap each other at a sink node, even if the RTS frames were sent to the sink node simultaneously by different sensor nodes. We consider that our underwater sink node can recover these nonoverlapping RTS frames. Since our RTS frame contains ID of the RTS sending node and a timestamp, the sink node calculates the propagation delay between the RTS sending node and the sink node, then broadcasts a CTS frame. Since our CTS frame contains the time information about when each RTS sending node can transmit a DATA frame to the sink node, multiple DATA frames transmitted by different sensor nodes can be formed as a train at the sink node. Then, the sink node broadcasts a BACK frame which contains whether each DATA frame was successfully received or not. We also proposed an underwater BACK protocol which is analogous to our proposed underwater multi-DATA train protocol except the multi-DATA train and the information contained in RTS and CTS frames. Then, we analyzed the normalized throughput and the mean access delay of our proposed underwater multi-DATA train protocol, our proposed underwater BACK protocol, the underwater multi-RTS reception protocol, and the conventional CSMA/CA protocol, respectively. The analytical results agree very well with the simulation results for throughput and delay performances of our proposed protocols and the conventional protocols, respectively, with various numbers of underwater sensor nodes and input parameters. The analytical results and the simulation results show that our proposed protocols outperform the conventional protocols. As a further study, we will extend our proposed protocols and our performance analysis to consider the scenario that underwater sensor nodes may be hidden from each other. The extension of our proposed protocols and our performance analysis will be helpful to study the scenario in UWSNs and IoUT.

## Figures and Tables

**Table 1:** Input parameters and values for analytical and simulation results.

Parameter	Value
Data rate $R_{\mathrm{UWA}}$	660 [bps]
Duration of PHY header $T_{\mathrm{PHY}}$	$128/R_{\mathrm{UWA}}$ [s]
Duration of MAC header $T_{\mathrm{MAC}}$	$272/R_{\mathrm{UWA}}$ [s]
Duration of RTS frame $T_{\mathrm{RTS}}$	$T_{\mathrm{PHY}}+\left(160/R_{\mathrm{UWA}}\right)$ [s]
Duration of CTS frame $T_{\mathrm{CTS}}$	$T_{\mathrm{PHY}}+\left(112/R_{\mathrm{UWA}}\right)$ [s]
Duration of DATA payload $T_{P}$	$8184/R_{\mathrm{UWA}}$ [s]
Duration of ACK frame $T_{\mathrm{ACK}}$	$T_{\mathrm{PHY}}+\left(112/R_{\mathrm{UWA}}\right)$ [s]
Duration of BACK frame $T_{\mathrm{BACK}}$	$T_{\mathrm{PHY}}+\left(112/R_{\mathrm{UWA}}\right)$ [s]
Underwater acoustic speed $v_{\mathrm{UWA}}$	1500 [m/s]
Cell radius $r_{C}$	2500 [m]
Maximum propagation delay ${\mathrm{PROP}}_{\max}$	$2r_{C}/v_{\mathrm{UWA}}$ [s]
SIFS	0.1 [s]
DIFS	${\mathrm{PROP}}_{\max}+\mathrm{SIFS}$ [s]
EIFS	$\mathrm{SIFS}+T_{\mathrm{ACK}}+\mathrm{DIFS}$ [s]
Slot time $T_{I}$	${\mathrm{PROP}}_{\max}$ [s]
Contention window size $W_{r}$	31, 63
